# Influence of Personal Social Network and Coping Skills on Risk for Suicidal Ideation in Chinese University Students

**DOI:** 10.1371/journal.pone.0121023

**Published:** 2015-03-24

**Authors:** Fang Tang, Ping Qin

**Affiliations:** 1 Department of Epidemiology and Biostatistics and Centre for Suicide Prevention and Research, School of Public Health, Shandong University, Jinan, China; 2 Health Management Centre, Qianfoshan Hospital Affiliated to Shandong University, Jinan, China; 3 National Centre for Suicide Research and Prevention, Institute of Clinical Medicine, University of Oslo, Oslo, Norway; Middlesex University Dubai, UNITED ARAB EMIRATES

## Abstract

**Background:**

Personal social network and coping skills have important influences on suicidality of young people and such influences must be understood in the context of other factors. This study aims to assess the influences of social contacts and coping skills on risk for suicidal ideation and to disentangle their possible pathways using a large sample of university students from China.

**Methods:**

5972 students, randomly selected from 6 universities in China, completed the questionnaire survey for the study. Logistic regression was performed to estimate individual effect of social contacts and coping skills on risk for suicidal ideation. A partial least squares path model (PLSPM) was used to probe possible paths of their effects in the context of psychopathology.

**Results:**

Of the 5972 students, 16.39% reported the presence of suicidal ideation. Poor social contacts were significantly associated with an increased risk for suicidal ideation. The influence of coping skills varied by coping styles adapted toward problems. A high score of skills on seeking guidance and support, problem solving as well as seeking alternative rewards was associated with a reduced risk of suicidal ideation; whereas a high score of acceptance or resignation, emotional discharge as well as logical analysis was associated with a significantly increased risk. Modeling the data with PLSPM indicated that the avoidance coping skills conferred the most important dimensional variable in suicidal ideation prediction, followed by the approach coping skills and social network.

**Conclusions:**

Poor social contacts and deficient coping skills are strong risk factors for suicidal ideation in young students. Prevention program focusing on these problems may have an enduring effect on reducing suicidal behavior in this population.

## Introduction

Suicidal ideation is a significant indicator for psychological problems in young people and may act as a predictor for suicide attempt and completion [1–4]. Among many factors associated with suicidal ideation in the young, personal social network and coping skills are of particular importance [5–11].

It has long been speculated that personal social network plays a critical role in the development of psychological distress and mental problems in young people [12–14]. Social contact with peers or friends to a large degree reflects a person’s psychological state and personality traits such as self-esteem, self-confidence, openness, etc. [7,13,15]. Previous studies have consistently indicated that people with mental or psychological problems tend to stay alone and exhibit withdrawal from social activities [16,17]. There is also convergent evidence linking poor social contacts with increased risks for suicidal behaviors, in particular for the development of suicidal ideation [15,18,19].

Coping is a process in which cognitive or behavioral efforts are made to manage specific internal and/or external sources of psychological stress [20,21]. In studying coping skills, researchers typically differentiate between approach and avoidant coping styles [22–25]. In general, approach coping is problem-focused and reflects cognitive and behavioral efforts to master or resolve life stressors. In contrast, avoidant coping tends to be emotion-focused and reflects cognitive and behavioral attempts to avoid thinking about a stressor and its implications, or managing the consequences induced by a stressor [25]. The importance of coping skills has been extensively explored in understanding suicide behaviors in young people [26,27], and a number of studies have shown a direct effect of coping skills on suicide risk [3,28–30]. More specifically, the use of emotion-focused and avoidant coping is reported to be associated with higher levels of suicidal ideation, whilst the use of problem-focused and approach coping is associated with fewer negative outcomes [26,31]. However, there are also controversies because some coping styles may be featured with both problem-focused and emotion-focused strategies, and the use of approach or avoidant coping may depend on the nature of a stressor and can even shift from one to another in everyday live [20].

Nevertheless, available studies on this topic have delved into the individual effect of social network or of coping skills on suicide risk; rarely has any study attempted to investigate the interplay of these two factors and to moreover elucidate possible underlying mechanisms of these factors on suicidality [16,19,32]. In the present study, we want to use data from a large sample of young university students to assess the relative importance of social contacts and coping skills on risk for suicidal ideation, and to probe their possible pathways of their effects in the context of personal psychopathology.

## Methods

### Selection of Study Subjects

Of 8 universities attached directly to the ministries of the P. R. China in Wuhan city, 6 universities agreed to join the survey for this study. A stratified cluster sampling method was used to draw a 10% sample of all undergraduate students in each university. The cluster of sampling was study class which is organized by specialty and school year with usually 50–120 students in each class. For each university, a list of study classes with information on specialty, school year and number of students was obtained from the university’s central academic administration office. Based on this list, study classes were drawn using numbers generated by the random function of a calculator until the cumulative number of students in the selected classes reached 10% of all undergraduate students in the university. Classes of specialties of medicine and psychology were excluded from the selection. In case a selected class had more than 100 students, 100 students were drawn randomly from such a class. Otherwise, all students were enrolled into the study. The rationale for restricting the number of participants to be selected from large clusters is to avoid the overweighting of big classes so that to ensure a better representativeness of drawn students to all students in the university. With this sampling procedure, a total of 7220 university students from 93 classes were selected as the study population and 6099 attended questionnaire survey for data collection, corresponding to a response rate of 84.4%. Following up of the 1121 students who did not attend the survey, indicated that most of these students were out of the university campus for their internship during the period when the survey was conducted whilst a small number of students chose not to participate in the study. Each enrolled student was assigned with an encrypted code unique to their student identification for the purpose of the survey.

The survey was conducted online via a website specifically designed for the present study. Access to the online questionnaires was restricted to students enrolled to the study using the unique encrypted code as password for login. All students were informed about the purpose of the study, the confidentiality of personal information and the principle of voluntary participation. For students who agreed to participate, signed consent forms were collected subsequently.

The survey started with an overall introduction about the purposes of the research, and then move on with online instructions to each specific questionnaire. Two pilot studies were carried out to examine the suitability and understandability of the questionnaires and a few pre-tests were also performed to test the functionality of the website. There was no report of technical problems during the final online survey collecting data from the students. 5972 university students completed all questionnaires designed for the present study and their data were therefore included in the analyses.

This study was approved by the Ethics Committee of Huazhong Normal University. The informed consents were obtained from all participants of the study.

### Measurements

Data on general characteristics such as sex, age and study specialty were collected from each participant. Suicidal ideation, social network, coping skills and psychopathology were assessed with self-designed or standard questionnaires as described below.

#### Suicidal Ideation

The dependent variable of suicidal ideation was assessed with two questions: (1) ‘‘Did you seriously think about committing suicide in the past 12 months?”, and (2) “Did you ever seriously think about committing suicide at any point in your lifetime?”. The questions were answered on a 3-point rating scale (0 = ‘never’, 1 = ‘sometimes’, 2 = ‘very often’). An answer of ‘1’ or ‘2’ to either of the two questions was regarded as presence of suicidal ideation.

#### Social Network

Personal social network referred to the students’ common social contacts and was assessed through 4 variables: number of close friends in the school, number of engaged group activities, status of a dating relationship and whether being the only child in the family. Students were asked to report the number of close friends in the school in categories of ‘none’, ‘1–2’, ‘3–5’, ‘5–10’, and ‘more than 10’. Number of engaged group activities was assessed through the question: “how many group activities do you participate in your spare time?” with the answer options of ‘none’, ‘1’, ‘2’, and ‘≥3’. A dating relationship and being the only child in the family were set as a binary variable with answer possibilities being ‘yes’ and ‘no’.

#### Coping Skills

The Coping Response Inventory (CRI), which is a 48-itemself-report instrument developed by Moos [25] to assess a person’s skills to cope with a wide variety of stressful life events, was used to evaluate the students’ coping skills. A Chinese version of the CRI was used to assess personal coping skills of the study population. The responses to this Likert-scaled instrument range from 0 (not at all) to 3 (fairly often) and the scale is made up from 8 subscales with 6 questions in each. 4 of the 8 subscales evaluate approach coping, i.e., Logical Analysis (LA), Positive Reappraisal (PR), Seeking Guidance and Support (SG) and Problem Solving (PS). The remaining four subscales evaluate avoidant coping, i.e., Cognitive Avoidance (CA), Acceptance or Resignation (AR), Seeking Alternative Rewards (SR) and Emotional Discharge (ED). The scoring was processed in accordance with the instruction manual of this instrument to facilitate comparisons among scales and with the normative group [25]. The direct sum scores of the 8 subscales were firstly converted to interpretive T scores and moreover categorized, according to the recommended cut-points in the manual [25], into 3 levels with values of 1 (T-score≤45), 2 (T-score 46–54) and 3 (T-score≥55), corresponding to the levels below average, average, and above average, respectively. In this study, the reliability of this instrument was high (Cronbach’s alpha = 0.92). It was also acceptable for overall approach coping (alpha = 0.91) and avoidance coping (alpha = 0.86) as well as for the 8 subscales (alpha range: 0.60–0.85).

#### Psychopathology

Psychopathology was assessed using the Chinese version of the Symptom Checklist-90-revised (SCL-90-R) [33]. The SCL-90-R measures participants’ self-reported psychopathologic features on nine subscales: somatization, obsessive-compulsiveness, interpersonal sensitivity, depression, anxiety, hostility, phobia, paranoid ideation, and psychoticism. Each question is rated on a five point Likert scale (0 for no distress, 4 for extreme distress). A higher score indicates a lower status of psychological health. In this study, the Cronbach’s alpha of this instrument was 0.95 and, for the nine subscales ranged from 0.82 (paranoid ideation) to 0.92 (depression). The nine symptom dimensions were included in the analysis primarily for the purposes of adjustment. The average score of each symptom dimension was calculated to reflect the status of psychopathologic features ranging from 0 to 4.

### Statistical Analysis

Descriptive analyses were performed to profile the distribution of variables of interest in the study population. Unconditional logistic regression analysis was used to estimate the individual effect of social contacts and coping skills on risk for suicidal ideation using the statistical package SAS, version 9.1. Students who answered positive to the presence of suicidal ideation were considered as the cases while those without suicidal ideation were used as the comparison controls. Crude odds ratios were only adjusted for gender. Adjusted odds ratios were further adjusted for psychopathology and all variables in the panel. Parallel analyses were also carried out separately for cases who reported having suicidal ideation “sometimes” and for those who reported having suicidal ideation “very often”.

In addition, a partial least squares path model (PLSPM) was used to probe possible pathways from social network and coping skills to suicidal ideation using software SmartPLS [34]. The PLSPM is a statistical approach for modeling complex multivariable relationships (structural equation models) among manifest (observed) and latent variables. It is a ‘soft modeling’ approach which requires very few distributional assumptions on variables of study [35]. The PLSPM modeling comprises an outer model and an inner model. The outer model relates manifest variables (MV) to each latent variable (LV) through a reflective mode or a formative mode. The inner model offers an explicit estimation of a network relationship of the latent variables [35]. In the outer model, a reflective mode (MV→LV) assumes that all MVs in the block are internally correlated, which would require tests for the homogeneity of these MVs and the unidimensionality of the block to be statistically satisfactory [37]. A formative mode (MV←LV) assumes that each manifest variable on the block represents a different dimension of the underlying concept and does not require the homogeneity nor unidimensionality of the block. For the model proposed in the present study, manifest variables related to the latent variable, on a satisfactory level to the PLSPM model requirements [35], through a reflective mode (MV→LV) for the blocks of approach coping, avoidance coping, psychopathology and suicidal ideation. While for the block of social network, manifest variables contributed to the block satisfactorily to the PLSPM model requirements through a formative mode (MV←LV), indicating that the manifest variables were independent and constructed this latent variable [35,37]. The path coefficients and loadings were estimated using Lohmöller algorithm, with significant test furnished by bootstrap procedures [35–38]. More details about the PLSPM could be found in the relevant references [35–38].

## Results

### Distribution and Prevalence

Of 5972 students included in this study 3191 were male (53.43%) and, 2781 were female (46.57%). The age of these students varied from 16 to 25 years old with the mean of 20.85 years old (SD: 0.58). 16.39% of the university students reported that they had ever seriously considered committing suicide, including a 15.82% of students who considered suicide sometimes and a 0.57% who considered suicide very often.

Table 1 and Table 2 show the descriptive statistics on explanatory variables according to the presence of suicidal ideation. Obviously, there were substantial differences between students with suicidal ideation and those without suicidal ideation. 20.35% female students reported the presence of suicidal ideation, which is significantly higher than the rate of 12.94% in male students (χ^2^ = 59.53, p<0.01). The rate was also higher among students who had relatively fewer close friends in the school, who attended relatively fewer group activities, and who did not have a dating relationship (Table 1). At the same time, compared with those without suicidal ideation, students with suicidal ideation had higher scores on avoidant coping subscales of cognitive avoidance, acceptance or resignation, and emotional discharge. Suicidal students also showed lower scores on approach coping subscales of seeking guidance and support, problem solving as well as avoidance coping skill of seeking alternative rewards. Moreover, suicidal students had significantly higher scores in all psychopathological symptoms measured through the SCL-90-R (Table 2).

**Table 1:** Descriptive statistics of categorical variables by presence of suicidal ideation in study population.

Variables		Suicidal ideation	No suicidal ideation	*x* ^2^ test
	N	N (%)	N (%)	
**Gender**				
Male	3191	413 (12.94)	2778 (87.06)	59.53**
Female	2781	566 (20.35)	2215 (79.65)	
**Number of close friends in the school**				
10 or above	575	55 (9.57)	520 (90.43)	126.83**
5~10	1022	123 (12.04)	899 (87.96)	
3~5	2774	425 (15.32)	2349 (84.68)	
1~2	1466	321 (21.90)	1145 (78.10)	
0	135	55 (40.74)	80 (59.26)	
**Number of engaged group activities**				
3 or above	593	85 (14.33)	508 (85.67)	16.06**
2	1706	270 (15.83)	1436 (84.17)	
1	2135	323 (15.13)	1812 (84.87)	
0	1538	301 (19.57)	1237 (80.43)	
**Status of dating relationship**				
In dating relation	979	247 (14.25)	1468 (85.75)	8.16**
No dating relation	4993	732 (17.27)	3507 (82.73)	
**Only child in the family**				
No	3732	610 (16.35)	3122 (83.65)	0.02
Yes	2240	369 (16.47)	1871 (83.53)	

**p<0.01.

**Table 2:** Descriptive statistics of continuous variables by presence of suicidal ideation in study population.

Variables	Mean (SD)		Cohen’s d	*t*-test
	Suicidal ideation (n = 979)	No suicidal ideation (n = 4993)		
**Approach coping skill**				
Logical analysis	50.90 (8.18)	51.17 (8.02)	-0.10	0.97
Positive reappraisal	52.55 (7.35)	52.80 (6.95)	-0.09	0.98
Seeking guidance and support	49.87 (8.12)	52.20 (8.13)	-0.82	8.18**
Problem solving	51.89 (8.71)	54.14 (8.50)	-0.77	7.56**
**Avoidance coping skill**				
Cognitive avoidance	54.73 (7.84)	53.28 (7.75)	0.52	-5.35**
Acceptance/resignation	52.17 (7.39)	49.70 (7.34)	0.91	-9.60**
Seeking alternative rewards	60.92 (7.67)	62.64 (7.50)	-0.63	6.51**
Emotional discharge	60.40 (8.62)	58.75 (9.00)	0.55	-5.29**
**Psychopathology (SCL-90-R)**				
Somatization	0.59 (0.66)	0.26 (0.37)	0.50	-15.10**
Obsessive-compulsiveness	1.19 (0.73)	0.68 (0.58)	0.65	-20.53**
Interpersonal sensitivity	1.06 (0.76)	0.52 (0.55)	0.70	-21.00**
Depression	0.99 (0.75)	0.44 (0.51)	0.74	-22.08**
Anxiety	0.88 (0.74)	0.39 (0.47)	0.68	-19.82**
Hostility	0.82 (0.74)	0.39 (0.49)	0.59	-17.29**
Phobia	0.66 (0.68)	0.28 (0.43)	0.55	-16.56**
Paranoid ideation	0.80 (0.71)	0.37 (0.46)	0.61	-18.28**
Psychoticism	0.81 (0.68)	0.36 (0.45)	0.64	-20.01**

**p<0.01.

### Risk of Suicidal Ideation Associated with Social Network and Coping Skills

Table 3 shows the odds ratios and corresponding 95% confidence intervals derived from the logistic regress model. Clearly, poor social network had a strong impact on risk for suicidal ideation. Compared with peer students with many close friends, the risk of suicidal ideation was significantly higher for students who had fewer close friends in the school—a tendency of the fewer friends the higher risk. Students who did not attend any group activities and students who did not have a dating relationship were also at a significantly increased risk for suicidal ideation. Being the single child in the family did not have a significant effect on risk for suicidal ideation. Meanwhile, relatively to the level of under average, higher levels of cognitive avoidance, acceptance or resignation and emotional discharge were associated with a significantly increased risk for suicidal ideation; whereas higher levels of seeking guidance and support, problem solving and seeking alternative rewards were associated with a significantly reduced risk.

**Table 3:** Risk of suicidal ideation associated with social network and coping skills.

Variables	Distribution		Risk for suicidal ideation (95%CI)	
	Suicidal cases (%)	Non-suicidal controls (%)	Crude odds ratio^a^	Adjusted odds ratio^b^
**Social network**				
**Number of close friends in the school**				
10 or above	5.62	10.41	1	1
5~10	12.56	18.01	1.22 (0.87–1.71)	1.15 (0.81–1.62)
3~5	43.41	47.05	1.53 (1.14–2.07) **	1.32 (0.97–1.80)
1~2	32.79	22.93	2.29 (1.68–3.12) **	1.75 (1.27–2.42) **
0	5.62	1.60	5.98 (3.84–9.34) **	2.94 (1.81–4.77) **
**Number of engaged group activities**				
3 or above	8.68	10.17	1	1
2	27.58	28.76	1.10 (0.84–1.43)	1.04 (0.79–1.37)
1	32.99	36.29	1.09 (0.84–1.41)	0.90 (0.69–1.19)
0	30.75	24.77	1.55 (1.19–2.02) **	1.15 (0.87–1.52)
**Status of dating relationship**				
In dating relation	25.23	29.40	1	1
No dating relation	74.77	70.24	1.29 (1.10–1.51) **	1.29 (1.09–1.52) **
**Only child in the family**				
No	62.31	62.53	1	1
Yes	37.69	37.47	0.98 (0.85–1.13)	0.98 (0.85–1.14)
**Coping skills**				
**Logical analysis**				
Under average	23.80	24.15	1	1
Average	37.69	36.83	1.04 (0.84–1.20)	1.36 (1.08–1.72) **
Above average	38.51	39.01	0.97 (0.81–1.15)	1.59 (1.21–2.09) **
**Positive reappraisal**				
Under average	16.45	15.38	1	1
Average	42.29	43.54	0.87 (0.71–1.06)	1.18 (0.90–1.55)
Above average	41.27	41.08	0.86 (0.70–1.05)	1.34 (0.98–1.84)
**Seeking guidance and support**				
Under average	31.26	21.73	1	1
Average	43.82	43.20	0.66 (0.56–0.78) **	0.63 (0.51–0.77) **
Above average	24.92	35.07	0.43 (0.35–0.52) **	0.37 (0.29–0.47) **
**Problem solving**				
Under average	26.25	19.01	1	1
Average	29.93	26.96	0.78 (0.65–0.99)*	0.87 (0.69–1.10)
Above average	43.82	54.04	0.56 (0.47–0.66) **	0.733 (0.57–0.95) *
**Cognitive avoidance**				
Under average	9.60	12.08	1	1
Average	38.10	44.68	1.04 (0.81–1.32)	0.84 (0.64–1.10)
Above average	52.30	43.24	1.41 (1.11–1.79) **	0.90 (0.67–1.20)
**Acceptance/resignation**				
Under average	17.57	29.56	1	1
Average	48.83	49.59	1.61 (1.34–1.94) **	1.52 (1.23–1.87) **
Above average	33.61	20.85	2.59 (2.12–3.17) **	2.24 (1.75–2.88) **
**Seeking alternative rewards**				
Under average	2.35	0.98	1	1
Average	16.55	12.68	0.49 (0.29–0.82) **	0.38 (0.20–0.73) **
Above average	81.10	86.34	0.34 (0.20–0.56) **	0.32 (0.17–0.60) **
**Emotional discharge**				
Under average	3.88	6.39	1	1
Average	25.74	31.66	1.25 (0.87–1.80)	1.62 (1.06–2.46) *
Above average	70.38	61.95	1.64 (1.16–2.33)**	1.95 (1.28–2.98) **

^a^: Crude odds ratios were adjusted for gender;

^b^: Adjusted odds ratios were further adjusted for psychopathologic features (SCL-90-R) and all variables in the panel simultaneously;

**p<0.01;

*p<0.05.

When all subtypes of coping skills and social contact variables as well as gender and psychopathologic features were considered simultaneously in the adjusted model (measure of the model fitness: R^2^ = 0.12), there were notable changes of the estimates associated with the variables under study. The risk effects of having few or no friends, no dating relationship, high levels of acceptance or resignation and of emotional discharge remained significant. High levels of skills on seeking guidance and support, problem solving, and seeking alternative rewards were still protective against suicidal ideation. High levels of logical analysis skill, however, became to have a risk effect on suicidal ideation in the full model.

Separate analyses for cases who reported having suicidal ideation “sometimes” and those who reported having suicidal ideation “very often” produced very consistent results, although the associated estimates for the late group were under powered because of too few cases (see S1 Table).

### Pathway Analysis using PLSPM model

**Fig 1:**
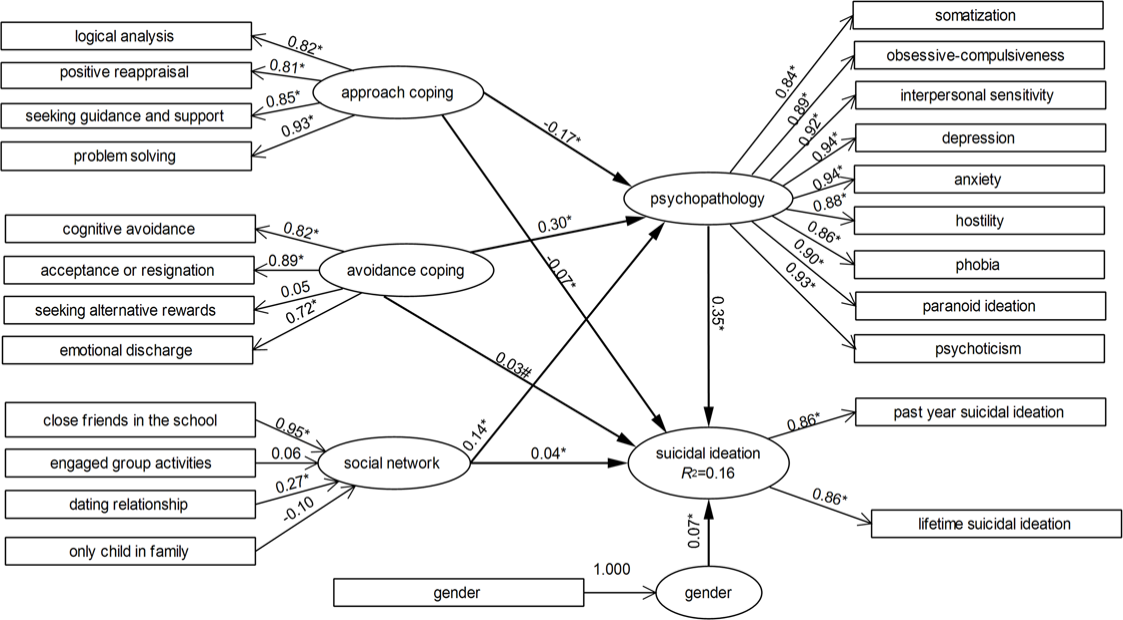
Structural model for social network and coping skills associated with suicidal ideation in study population. *p<0.05.

Fig. 1 shows the results from the final PLSPM model that illustrated possible pathways of social network and coping skills on suicidal ideation. The determination coefficient of the model (R^2^) was 0.16, indicating that the model explained 16% of the variance in the data and thus had an acceptable capacity in explanation of suicidal ideation.

For the outer model of the block of Social Network, the results showed that the manifest variable of number of close friends in the school was the most significant contributor in the formation of Social Network (loading = 0.95, t = 43.38) followed by the status of dating relationship (loading = 0.27, t = 4.68); while attendance of group activities and being the only child in the family contributed insignificant to the block. For the block of Avoidance Coping, acceptance or resignation was the most important contributor (loading = 0.89, t = 86.52), followed by cognitive avoidance (loading = 0.82, t = 46.92). The skill of seeking alternative rewards was not a significant contributor to this block, probably because this coping skill had a protective effect against suicidal ideation. For the block of Approach Coping, problem solving was the most important contributor (loading = 0.93, t = 146.44), followed by seeking guidance and support (loading = 0.85, t = 101.81). Moreover, all measurement variables contributed significantly in the blocks of Psychopathology and Suicidal Ideation.

For the inner model, Social Network, Approach Coping and Avoidance Coping all had a direct effect as well as some indirect effect on Suicidal Ideation through Psychopathology. The total effect of each latent variable (β) was the sum of its direct and indirect effects on Suicidal Ideation. More specifically, except for the known prominent effect of Psychopathology, Avoidance Coping was the most important risk factor [β = 0.14, including a direct effect of 0.03, and an indirect effect of 0.11 (0.30×0.35 = 0.11)] in the prediction of Suicidal Ideation, followed by poor Social Network (β = 0.09, direct effect: 0.04, indirect effect: 0.14×0.35 = 0.05). The Approach Coping was negatively associated with Suicidal Ideation (β = -0.13, direct effect: -0.07, indirect effect: -0.17×0.35 = -0.06).

## Discussion

### Findings and Explanations

This large study demonstrates that, 16.39% of 5972 students enrolled in the study reported the presence of suicidal ideation at some point of their lifetime. Poor social contacts were significantly associated with an increased risk for suicidal ideation. The influence of coping skills varied according to the coping styles adapted toward stressful events or circumstances. A high score of skills on seeking guidance and support, problem solving as well as seeking alternative rewards was associated with a reduced risk of suicidal ideation; whereas a high score of acceptance or resignation, emotional discharge and logical analysis was associated with a significantly increased risk. Modeling the data with PLSPM indicated that the avoidance coping skill conferred the most important dimensional variable in the prediction of suicidal ideation, followed by the approach coping skills and social network.

Our finding of 16.39% students with suicide ideation is in line with the rates of 20–30% found in previous studies based on smaller samples of young people in China [3,4,40] and also with studies from other countries [41–44]. The observed gender difference in the prevalence of suicidal ideation in our study population is consistent with other studies reporting that females were more likely to report suicidal ideation [40,45–47].

Consistent with previous studies [10,16,19,32], this study shows that risk of suicidal ideation was highly associated with poor social network, reflected by fewer close friends and without a dating relationship. It is a known fact that for young people friends are an important source of support when needed [7]. Young people are usually more willing to talk with friends, than family members, about their mental state and behavior without the concern of being judged. They are also more prone to share personal feelings and thoughts with their peer friends than with the families. The support and comfort attained from network friends could help them dealing with critical situation and stressors effectively and in a positive way. Our findings indicate the importance of the “social influences” approach [16] in suicide prevention. Efforts encouraging students, especially those who stay isolated, to be more active in social contacts and group activities may have a significant beneficial effect in suicide prevention and mental health care.

In regard to coping skills, the present study demonstrates a generally protective effect of approach coping and a risk effect of avoidant coping, but also indicates that the influence of coping skills can vary significantly by specific subscales regardless whether they are approach or avoidance coping styles. For instance, seeking alternative rewards is regarded as avoidance coping but has a protective effect against suicidal ideation, while logical analysis is viewed as approach coping but is associated with an increased risk of suicidal ideation when adjusted for other types of coping skills. Yet people may apply coping styles differently according to types of stress or event [25], our results suggest that, it is beneficiary to have high skills of seeking guidance and support, problem solving and seeking alternative rewards, rather than overreliance on thinking about a problem (such as logical analysis), resignation, and even passive acceptance. In the context of Chinese society, young people are often overprotected by their parents and not well prepared for dealing with stressful situations or circumstances when they move away from home to live in university campuses. Therefore, improvement of their coping skills could be an effective way to enhance their mental well-being during university life and also to prevent them from thinking about suicide when encountering setbacks.

Our pathway analysis indicates that social network, approach coping and avoidance coping have direct effects as well as indirect effects on suicidal ideation through psychopathology. In the pathways from social network to suicidal ideation, the indirect effect is slightly larger than the direct effect; from avoidance coping to suicidal ideation, the indirect effect contributes a larger proportion; but from approach coping to suicidal ideation, the direct effect accounts for a larger proportion. These findings provide valuable insights for strategies of suicide prevention. Programs targeting on expansion of social network and improvement of approach coping skills may have enduring effects on reducing suicidal ideation in this group of young people.

### Strengths and Limitations

A major strength of the present study is the large sample of population that was randomly drawn from 6 comprehensive universities in China, making this study insofar the largest one to study suicidal behavior among Chinese university students. Another strength is that variables of study include a range of social network indicators and coping skills measurements. This enables an insightful understanding of their influences and pathways on suicidal ideation. Moreover, the use of PLSPM to model our data has some advantages in itself. Specifically, the PLSPM requires very few distributional assumptions. For instance, the variables included in the model can be numerical, ordinal or nominal, and do not need to have a normal distribution in the study population. This makes the model more feasible when compared with ‘hard modeling’ approaches, such as covariance-based Structural Equation Model (SEM), that usually require heavy distributional assumptions [35,36,39].

The study also bears several limitations. Our measures of social network mainly covered some school-related social contacts, leaving social connections outside school unmeasured and thus unconsidered in the analyses. Also, our results might be affected by social desirability bias because of the nature of survey by self-report and because suicidal behavior is often regarded undesirable in young people especially in Chinese culture. Moreover, the pathways from social network and coping skills to suicidal ideation are more complicated than the model proposed in this study, but were not tested with other models in comparison with the PLSPM. Additionally, the present study is a cross-sectional investigation, which makes it impossible to demonstrate a causal relationship of the outcome with the exposures. The study subjects solely consisted of undergraduate students, which may limit the generalizability of our findings to all young people in contemporary China.

Nevertheless, this large study demonstrates that suicidal ideation among university students is highly associated with their social network and coping skills, and that the influence of these factors can be manifested through a direct effect and an indirect effect via psychopathological traits. Efforts of suicide prevention should include programs or activities that can extend students’ social contacts, increase various coping skills and enhance students’ capability of applying appropriate coping strategies towards specific stressors. Such efforts should be integrated into the existing programs for mental health care in the university settings.

## Supporting Information

S1 Table Risk of suicidal ideation associated with social network and coping skills, stratified by frequency of having suicidal ideation. ^a^: Adjusted odds ratio were adjusted for gender, psychopathologic features (SCL-90) and all variables in the table simultaneously; **p<0.01; *p<0.05.
(DOCX)
