# Supplementary material for: Influence of Personal Social Network and Coping Skills on Risk for Suicidal Ideation in Chinese University Students
Source: PLoS One. 2015 Mar 24;10(3):e0121023. doi: 10.1371/journal.pone.0121023 (PMC4372485; doi:10.1371/journal.pone.0121023)
Supplement: S1 Table — a: Adjusted odds ratio were adjusted for gender, psychopathologic features (SCL-90) and all variables in the table simultaneously; **p<0.01; *p<0.05. (DOCX) [file pone.0121023.s001.docx]

**Supplement Table**

**Risk of suicidal ideation associated with social network and coping skills, stratified by frequency of having suicidal ideation**

| **Variables** | **Distribution, n (%)** | | |  | **Adjusted odds ratio^a^ (95%CI)** | |
| --- | --- | --- | --- | --- | --- | --- |
|  | **No Suicidal Ideation** | **Suicidal Ideation sometimes** | **Suicidal Ideation**  **very often** |  | **Suicidal ideation**  **sometimes** | **Suicidal ideation**  **very often** |
| **Number of close friends in the school** |  |  |  |  |  |  |
| 10 or above | 520 (10.41) | 54 (5.71) | 1 (2.94) |  | 1.00 | 1.00 |
| 5~10 | 899 (18.01) | 117 (12.38) | 6 (17.65) |  | 1.09(0.77-1.55) | 9.56(0.32-283.15) |
| 3~5 | 2349 (47.05) | 415 (43.92) | 10 (29.41) |  | 1.29(0.95-1.76) | 4.46(0.16-125.95) |
| 1~2 | 1145 (22.93) | 311 (32.91) | 10 (29.41) |  | 1.72(1.24-2.37)^**^ | 6.71(0.23-199.18) |
| 0 | 80 (1.60) | 48 (5.08) | 7 (20.59) |  | 2.77(1.69-4.53)^**^ | 15.49(0.44-545.12) |
| **Number of engaged group activities** |  |  |  |  |  |  |
| 3 or above | 508 (10.17) | 82 (8.68) | 3 (8.82) |  | 1.00 | 1.00 |
| 2 | 1436 (28.76) | 263 (27.83) | 7 (20.59) |  | 1.04(0.78-1.37) | 1.44(0.22-9.32) |
| 1 | 1812 (36.29) | 312 (33.02) | 11 (32.35) |  | 0.90(0.68-1.18) | 1.61(0.27-9.73) |
| 0 | 1237 (24.77) | 288 (30.48) | 13 (38.24) |  | 1.14(0.86-1.52) | 2.16(0.36-12.83) |
| **Status of dating relationship** |  |  |  |  |  |  |
| In dating relation | 1486 (29.76) | 237 (25.08) | 10 (29.41) |  | 1.00 | 1.00 |
| No dating relation | 3507 (70.24) | 708 (74.92) | 24 (70.59) |  | 1.30(1.10-1.54)^**^ | 1.00(0.42-2.41) |
| **Being the only child in the family** |  |  |  |  |  |  |
| No | 3122 (62.53) | 594 (62.86) | 16 (47.06) |  | 1.00 | 1.00 |
| Yes | 1871 (37.47) | 351 (37.14) | 18 (52.94) |  | 0.97(0.83-1.13) | 1.92(0.85-4.37) |
| **Approach coping skill** |  |  |  |  |  |  |
| **Logical analysis** |  |  |  |  |  |  |
| Under average | 1206 (24.15) | 223 (23.60) | 10 (29.41) |  | 1.00 | 1.00 |
| Average | 1839 (36.83) | 358 (37.88) | 11 (32.35) |  | 1.34(1.06-1.69)^*^ | 4.51(0.92-22.22) |
| Above average | 1948 (39.01) | 364 (38.52) | 13 (38.24) |  | 1.56(1.19-2.05)^**^ | 4.06(0.67-24.46) |
| **Positive reappraisal** |  |  |  |  |  |  |
| Under average | 768 (15.38) | 149 (15.77) | 12 (35.29) |  | 1.00 | 1.00 |
| Average | 2174 (43.54) | 408 (43.17) | 6 (17.65) |  | 1.22(0.92-1.60) | 0.13(0.03-0.66)^*^ |
| Above average | 2051 (41.08) | 388 (41.06) | 16 (47.06) |  | 1.36(0.98-1.87) | 0.52(0.11-2.50) |
| **Seeking guidance and support** |  |  |  |  |  |  |
| Under average | 1085 (21.73) | 294 (31.11) | 12 (35.29) |  | 1.00 | 1.00 |
| Average | 2157 (43.20) | 414 (43.81) | 15 (44.12) |  | 0.62(0.50-0.76)^**^ | 1.05(0.28-4.05) |
| Above average | 1751 (35.07) | 237 (25.08) | 7 (20.59) |  | 0.37(0.29-0.48)^**^ | 0.37(0.07-1.89) |
| **Problem solving** |  |  |  |  |  |  |
| Under average | 949 (19.01) | 243 (25.71) | 14 (41.18) |  | 1.00 | 1.00 |
| Average | 1346 (26.96) | 285 (30.16) | 8 (23.53) |  | 0.86(0.68-1.09) | 0.86(0.22-3.37) |
| Above average | 2698 (54.04) | 417 (44.13) | 12 (35.29) |  | 0.73(0.56-0.94)^*^ | 0.95(0.22-4.01) |
| **Avoidance coping skill** |  |  |  |  |  |  |
| **Cognitive avoidance** |  |  |  |  |  |  |
| Under average | 603 (12.08) | 86 (9.10) | 8 (23.53) |  | 1.00 | 1.00 |
| Average | 2231 (44.68) | 367 (38.84) | 6 (17.65) |  | 0.85(0.64-1.12) | 0.25(0.04-1.53) |
| Above average | 2159 (43.24) | 492 (52.06) | 20 (58.82) |  | 0.90(0.67-1.22) | 0.66(0.12-3.75) |
| **Acceptance or resignation** |  |  |  |  |  |  |
| Under average | 1476 (29.56) | 163 (17.25) | 9 (26.47) |  | 1.00 | 1.00 |
| Average | 2476 (49.59) | 471 (49.84) | 7 (20.59) |  | 1.54(1.25-1.90)^**^ | 0.96(0.21-4.35) |
| Above average | 1041 (20.85) | 311 (32.91) | 18 (52.94) |  | 2.22(1.73-2.86)^**^ | 3.67(0.78-17.34) |
| **Seeking alternative rewards** |  |  |  |  |  |  |
| Under average | 49 (0.98) | 16 (1.69) | 7 (20.59) |  | 1.00 | 1.00 |
| Average | 633 (12.68) | 156 (16.51) | 6 (17.65) |  | 0.48(0.25-0.96)^*^ | 0.05(0.01-0.44)^**^ |
| Above average | 4311 (86.34) | 773 (81.80) | 21 (61.76) |  | 0.40(0.20-0.80)^*^ | 0.03(0.00-0.27)^**^ |
| **Emotional discharge** |  |  |  |  |  |  |
| Under average | 319 (6.39) | 32 (3.39) | 6 (17.65) |  | 1.00 | 1.00 |
| Average | 1581 (31.66) | 249 (26.35) | 3 (8.82) |  | 1.63(1.06-2.50)^*^ | 0.75(0.07-7.70) |
| Above average | 3093 (61.95) | 664 (70.26) | 25 (73.53) |  | 1.94(1.26-2.96)^**^ | 2.64(0.29-24.13) |

^a^: Adjusted odds ratio were adjusted for gender, psychopathologic features (SCL-90) and all variables in the table simultaneously; ^**^p<0.01; ^*^p<0.05
